# Single cell analysis applied to antibody fragment production with *Bacillus megaterium*: development of advanced physiology and bioprocess state estimation tools

**DOI:** 10.1186/1475-2859-10-23

**Published:** 2011-04-15

**Authors:** Florian David, Antje Berger, Robert Hänsch, Manfred Rohde, Ezequiel Franco-Lara

**Affiliations:** 1 Institute of Biochemical Engineering, Technische Universität Braunschweig. Gausstrasse 17, 38106 Braunschweig, Germany; 2 Botanical Institute, Technische Universität Braunschweig. Humboldtstraße 1, 38106 Braunschweig, Germany; 3 Helmholtz Centre for Infection Research Inhoffenstraße 7, 38124 Braunschweig, Germany

## Abstract

**Background:**

Single cell analysis for bioprocess monitoring is an important tool to gain deeper insights into particular cell behavior and population dynamics of production processes and can be very useful for discrimination of the real bottleneck between product biosynthesis and secretion, respectively.

**Results:**

Here different dyes for viability estimation considering membrane potential (DiOC_2_(3), DiBAC_4_(3), DiOC_6_(3)) and cell integrity (DiBAC_4_(3)/PI, Syto9/PI) were successfully evaluated for *Bacillus megaterium *cell characterization. It was possible to establish an appropriate assay to measure the production intensities of single cells revealing certain product secretion dynamics. Methods were tested regarding their sensitivity by evaluating fluorescence surface density and fluorescent specific concentration in relation to the electronic cell volume. The assays established were applied at different stages of a bioprocess where the antibody fragment D1.3 scFv production and secretion by *B. megaterium *was studied.

**Conclusions:**

It was possible to distinguish between live, metabolic active, depolarized, dormant, and dead cells and to discriminate between high and low productive cells. The methods were shown to be suitable tools for process monitoring at single cell level allowing a better process understanding, increasing robustness and forming a firm basis for physiology-based analysis and optimization with the general application for bioprocess development.

## Background

Flow cytometry was originally established for characterizing and sorting mammalian cells but won recently more and more importance applied at microbial processes [1,2], medical applications [3-5], dairy industry [6,7], alcoholic beverage production [8] and environmental and water systems [9]. In industrial production processes, single cell analysis may give high resolution insights into whole cell cultures concerning the cell status of viability, metabolic activity or even productivity [1,10].

Considering production processes in biotechnological industry, Process Analytical Technologies (PAT) for monitoring and evaluation of these processes is gaining more and more importance [11]. Here one main point is to easily decide whether a process is stable and reliable in production according to Food and Drug Administration (FDA) standards. Its main idea is to move away from quasi-final, off-line product quality assessment to "real time" strategies accompanied by online measurements of critical variables. To gain a better understanding and deeper insights into single cell performances, appropriate methods to characterize the physiology of a particular bacterial population have to be developed. Therefore adapted staining and measurement protocols considering the suitability of dyes, concentration and incubation times are necessary. Here it is important to verify if the used method is specific and sensitive enough to measure slight changes in the physiological parameter investigated leading to quantitative and not only to qualitative results. Especially for bioreactor cultivation with changing cell properties over time [12-14], the robustness and applicability of a method should be assured in order to consider it suitable as appropriate at line technique for process monitoring.

In the underlying case, *B. megaterium *producing and secreting antibody fragment (ABF) D1.3 scFv, a lysozyme specific model antibody fragment, was in focus of investigations on single cell level for bioprocess control. *B. megaterium *is a Gram positive bacterium with high secretion capacities [15] and was shown to efficiently secrete proteins like penicillin G amidase [16], levansucrase [17] and hydrolase from *Thermobifida fusca *[18]. Besides these recombinant proteins, antibody fragments also were proven to be effectively secreted into the supernatant [19]. Antibodies and antibody fragments are important tools in therapy and diagnostics [20-22]. The aim in using microbial cells to express them, is to have high effective, time and money saving production and secretion systems for a high value product. Thereby the most cost intensive downstream processing can be reduced regarding the separation time and may be realized by one step purification methods, e.g. by Immobilized Metal Affinity Chromatography (IMAC) or Protein-L purification (©Pierce).

Recently, a minimal medium of high production and secretion capacities could be developed based on a genetic algorithm approach varying metal ion concentrations [23]. Based on this minimal medium, batch cultivations were carried out and single cell analysis under controlled bioprocess conditions was performed to study the D1.3 scFv ABF production with *B. megaterium *at different stages of the cultivation.

For microbial bioprocess development it is most important to monitor cell stress response related to cell viability as such information determines the process efficiency [24]. This can be done by cell integrity and membrane potential (MP) measurements. The MP is primary coupled to the [H^+^] gradient of a cell, which is itself a direct parameter for the essential ATP generation capacity. The underlying [H^+^] gradient is generated by the electron transport through the electron transport chain being related to NADH_2 _availability and is directly associated with the metabolic activity of every single cell. Changes in MP are therefore the most sensitive indicators for culture status estimation whereas cell integrity measurements relate to more distinctive parameters like compromised cell membrane and cell wall structures.

A well established, accurate and high resolving method for measuring MPs is therefore an ideal instrument to either immediately adapt process conditions by means of process control or to abort batch processes in early stages. In addition, unstable MPs and development of heterogeneities can be regarded as key variables to deduce information about the process robustness itself, assumed the methods used are sensitive enough to conclude qualitative information.

Dyes for estimation of MP and cell integrity assays should be chosen very carefully as they may change cell properties themselves, only work on artificially created negative controls, e.g. heat treatment, and might therefore not be suitable for at line bioprocess control.

For cell status estimation considering MP and cell integrity measurements, diverse approaches were followed. The underlying MP was estimated by evaluating different fluorescent probes of 3,3'-diethyloxacarbocyanine iodide (DiOC_2_(3)) [25], 3,30-dihexyloxacarbocyanine iodide (DiOC_6_(3)) [24] and bis-(1,3-dibutylbarbituric acid) trimethine oxonol (DiBAC_4_(3)[26]). Here DiOC_6_(3) and DiOC_2_(3) are positively charged lipophilic fluorescent probes binding to the membrane of actively growing cells and were successfully used for Gram positive bacteria before [27]. DiBAC_4_(3) shows lipophilic properties, has an anionic charge and therefore is not able to pass through polarized membranes of living bacteria. Instead it was shown to enter cells at membrane potential depletion and binds to positively charged proteins or less specifically to hydrophobic regions. For cell integrity measurements protocols using propidium iodide (PI) as potential probe for live dead/measurements [28] combined either with Syto9 or DiBAC_4_(3) were evaluated.

Additional process relevant methods are those focusing on discriminating information related to single cell productivity, i.e. on developing reliable methods to distinguish between high, low and non-producing cells. Here the general question might be answered if an underlying reduced productivity is due to less effective production in every single cell of a homogeneous population or may be due to differentiated cell population of producing and non-producing cells. Under normal circumstances only average values are taken into account. Especially for high cell density cultivations, population analysis may lead to totally new insights into reduced specific productivities. By single cell analysis of secretion processes one may find out where the actual bottleneck of production and/or secretion is located.

To gain deeper insights into the single cell production status of ABF producing *B. megaterium *a fluorescence intensity based detection assay was newly developed. This method is based on detection antibodies binding at the secreted functionally folded ABF stick to the cell. Thereby the fluorescence of cells is increased due to specific labeling of high producing/secreting cells by Alexa Fluor 488 coupled detection antibodies. The method was evaluated by means of Confocal Laser Scanning Microscopy (CLSM) and Transmission Electron Microscopy (Immuno-FESEM) and applied on a bioreactor cultivation.

## Results

### Dye validation

Different dyes used to estimate MP measurements like DiOC_2_(3), DiOC_6_(3), DiBAC_4_(3) were tested regarding applicability with optimized concentration and incubation times (Table 1). MPs were evaluated according to the maximal signal difference between carbonyl cyanide m-chlorophenylhydrazone (CCCP) treated cells from exponential phase (control for depolarized cells) [25] and exponential growing cells (polarized) at particular combinations of incubation times and applied dye concentrations. The higher the difference the more precise is the final resolution of MP estimations. Here the resulting mean values of the specific fluorescence concentrations (FL-FC) of particular fluorescence distributions or regions were taken into account. In the underlying dye evaluation DiOC_2_(3) and DiOC_6_(3) as cyanine dyes were shown to increasingly stain polarized cells whereas DiBAC_4_(3) instead stains predominantly depolarized cells. At both cases, the particular difference in fluorescence intensity of exponential growing and depolarized (CCCP treated) cells was determined.

**Table 1:** Dye Screening for MP estimation

Dye	Increasing fluorescence according to cell status	Concentration range (μM)	Incubation time range (min)	Maximum difference to depolarized cells with CCCP		Influence on	
				
				Concentration (μM)	Incubation time t (min)	SS (% ± SD)	EV (% ± SD)
**DiOC**_**2**_**(3)**	Red (675 nm)/green (525 nm) ratio, polarized	4-80	0-43	14	5 < t < 27	-18 (±8)	-19 (±5)

**DiOC**_**6**_**(3)**	Green (525 nm), polarized	0.015-0.4	0-36	0.365	5 < t <28	-14 (±6)	-54 (±5)

**DiBAC**_**4**_**(3)**	Green (675 nm), depolarized	0.02-3	0-43	2.1	2 < t <4	-47 (±8)	-84 (±8)

Overview of different dyes used to estimate MP of *B. megaterium *cells, optimized concentration ranges and incubation times with their particular effect on Side Scatter (SS) and Electronic Volume (EV) measurements.

Interestingly, the signal intensity over time was stable for DiOC_2_(3) and DiOC_6_(3) staining (5 < t < 28 minutes) whereas applying DiBAC_4_(3) the staining of depolarized cells decreased over incubation time and remained stable only at a short time period (2 < t < 4 minutes). This may be due to the different chemical properties of DiBAC_4_(3) as oxonol stain (negatively charged) compared to DiOC_2_(3) and DiOC_6_(3), which are carbocyanides (positively charged) [25]. Optimal dye concentrations and incubation times were tested in bioreactor cultivations with multiple sample points throughout different growth phases. The effects on Side Scatter (SS) signal, respectively the granularity of the cell and electronic volume (EV), shown in table 1, were estimated from a bioreactor cultivation where the SS and EV values of all dyed samples were referred to non-dyed samples. Here a mean value of the particular difference and its standard deviation were determined. This direct application revealed dye depended effects on single cell properties like the EV and SS. Although DiBAC_4 _(3) and DiOC_6_(3) were most efficient in distinguishing polarized from depolarized (CCCP treated) cells, the dyes showed no explicit application on bioreactor cultivations samples. This may be due to the fact that both dyes change SS and EV signals up to 84% leading to non-significant measurements. On the contrary, DiOC_2_(3) could be easily applied to estimate MP and was at the same time the dye with minimal influence on single cell properties like EV and SS. Therefore this dye was the ideal choose for MP estimation.

### MP estimation

**Figure 1:**
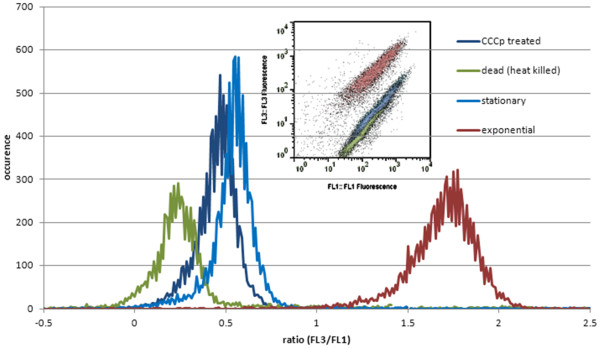
**Membrane Potential (MP) estimation**. MP measurements of *B. megaterium *cells producing antibody fragment scFv D1.3. DiOC_2_(3) staining and subsequent FL3/FL1 ratio analysis regarding different growth stages (exponential and stationary) and treatment with heat and CCCP as negative controls.

Figure 1 illustrates the reproducible measurements of DiOC_2_(3) stained cells evaluated according the FL3/FL1 ratio method described in material and method section. Cells from exponential phase were measured in comparison to dead, stationary and CCCP treated exponential phase cells, the late used as a control for depolarized cells. Here it is assumed that DiOC_2_(3) enters polarized cells in such an amount, that the normally green fluorescence dye showed increased red fluorescence properties due to agglomeration of dye molecules [25]. Therefore an increased red fluorescence directly reflects the state of optimal membrane polarization and the enhanced metabolic activity of the corresponding cell population. By dividing through the green fluorescence value, normalization is done according to the cell size. Here fluorescence distributions from stationary phase cells are lying in the same area like CCCP treated cells reflecting the applicability of this staining method to estimate MPs. Moreover, the fluorescence distributions (FL3/FL1 ratios) of polarized and depolarized cells are clearly separated illustrating the high sensitivity level.

DiOC_2_(3) was chosen for the following experiments as the optimal dye to estimate MPs with indicated concentration and incubation times guaranteeing sensitive and robust measurements. As no real MP has been estimated for *B. megaterium* so far, a calibration between the measured fluorescence intensities and the existing MPs was performed.

**Figure 2:**
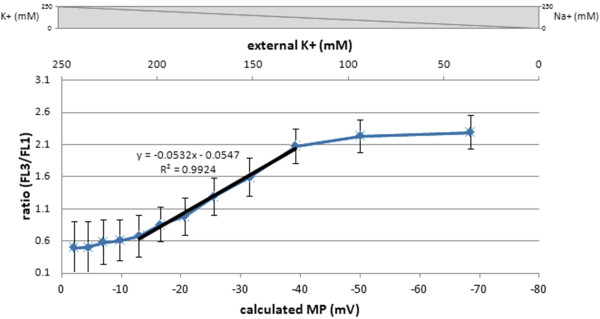
**MP calibration**. Calibration of MP related to DiOC_2_(3) stain FL3/FL1 ratio analysis of *B. megaterium *cells producing antibody fragment scFv D1.3. MP was simulated by Valinomycin and potassium addition and calculated by the Nernst equation. Error bars representing coefficient of variation CV values of particular FL3/FL1 distributions.

The calibration with different potassium ion concentrations [K^+^] accompanied with valinomycin treatment was carried out to artificially simulate certain MPs according to the method of Novo *et al*. (1999). The calculated MPs were correlated to measured DiCO_2_(3) stain intensities of *B. megaterium *cells producing antibody fragment D1.3 scFv. From the applied [K^+^] concentrations, the particular MP was determined using the Nernst equation and related to the measured FL3/FL1 ratio values of particular samples. Here, a strong linear correlation could be observed in a wide range with saturation values on both sides of polarized and depolarized cells showing a sigmoidal curve relationship (Figure 2). A maximal polarization of the membrane of -50 mV could be determined which may be due to the fact that ABF producing cells were measured (other cell types showed MP of -120 mV, but without heterologous protein production and secretion [29]). The relatively low MP calculated might also be due to a toxic effect of the dye itself as no distinctive hyperpolarization at low [K+] concentrations could be measured under valinomycin treatment. Despite this, the observed linear correlation clearly shows that the staining intensity of DiOC_2_(3) is directly correlated to the MP created by the applied [K^+^] concentrations which again highlights the sensitivity and applicability of the method.

### Cell integrity estimation

**Figure 3:**
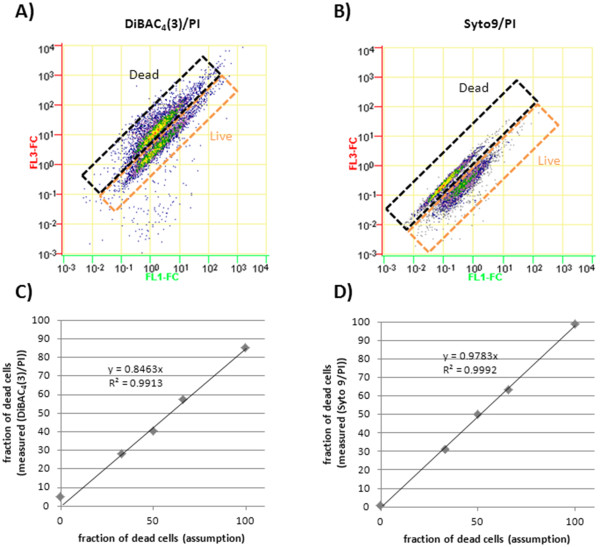
**Viability estimation**. Live/dead test of *B. megaterium *cells producing ABF D1.3 scFv with different dye combinations of DiBAC_4_(3)/PI and Syto9/PI. **A) **DiBAC_4_(3)/PI stain: 50% dead cell, 50% live cell mixture, **B) **Syto9/PI stain: 50% dead cell, 50% live cell mixture. **C)**, **D) **Calibration curves were determined via different mixtures of dead (heat killed) and live (exponential phase) cells.

Apart from MP estimation, the cell integrity is also an important parameter for bioprocess evaluation especially during long term starvation periods. Here the differentiation between dormant depolarized cells and dead cells indicated by compromised cell membrane is most desirable. Dye combinations of Syto9/PI and DiBAC_4_(3)/PI were tested on *B. megaterium *cells which were heat killed and/or taken from exponential growth phase (Figure 3). Different mixtures of these cells were investigated and could be directly correlated to the resulting clusters representing the differentiated populations. Figure 3 clearly shows the applicability of both dye combinations at correlated data. Here the fluorescence concentration was considered to ensure accurate measurements of florescence intensity related to the particular cell volume.

At both dye combinations an increase of red fluorescence in dead cells was expected as PI should be able to enter the cells and bind to nucleic acids, thereby increasing in fluorescence intensity. Heat killed cells were expected to show a higher green fluorescent due to DiBAC_4_(3) staining related to the depolarized MP. However at both measurements PI may have led to a quenching of green fluorescence of Syto9 and DiBAC_4_(3), respectively. The predominant reduction of green fluorescence of heat killed cells at the Syto9/PI assay may also be related to the displacement of Syto9 by PI, which enters the cells in this non vital cell status competing for the same binding at nucleic acid sites.

### Production intensity

**Figure 4:**
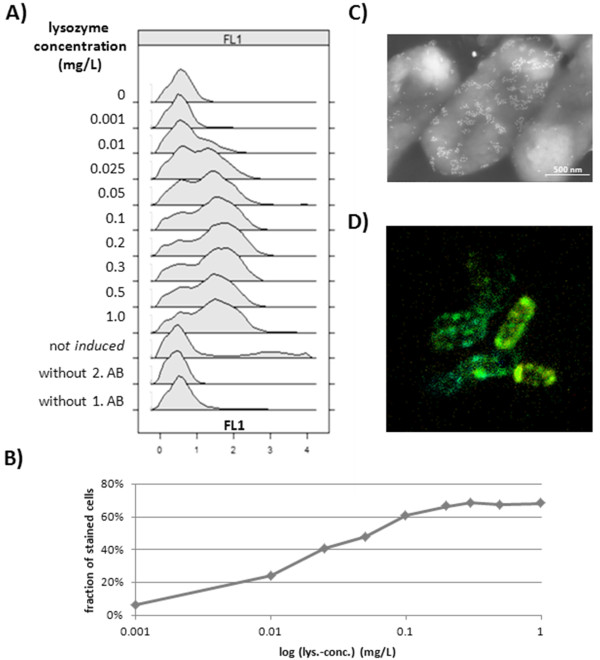
**Production intensity assay-validation**. Lysozyme treatment of *B. megaterium *cells producing antibody fragment D1.3 scFv for assay evaluation distinguishing between producing and non-producing cells. **A)**, **B) **different lysozyme concentrations and their effect on staining intensity with second antibody (AB) Alexa Fluor 488. **C) **Immuno-FESEM picture of ABF D1.3 scFv secreting, lysozyme treated *B. megaterium *cells incubated with a second gold coupled detection antibody. **D**) Confocal Laser Scanning Microscopy (CLSM) picture of *B. megaterium *cells, lysozyme treated combined with Alexa Fluor 488 as second detection antibody.

Especially in biotechnology applications concerning heterologous protein production the specific productivity at single cell level is an important process variable. Therefore an assay to measure this productivity status and distinguish between ABF D1.3 scFv producing/secreting and non-producing/non-secreting *B. megaterium *cells was developed. By first fixing cells with paraformaldehyde, the ABF D1.3 scFv secreted through the cell membrane sticks to the bacterial cell surface and becomes measurable by detection antibodies. In this case a first anti-penta His antibody was used to detect the His-tag of the secreted antibody fragment, and a second anti mouse antibody coupled with the fluorochrome Alexa Fluor 488 was used to specific label ABF D1.3 scFv producing cells and make them observable for flow cytometry and CLSM analysis. As shown in figure 4A, the lysozyme treatment is indispensable to make the ABF fragment reachable for detection antibodies. In this experimental screening with different lysozyme concentrations not all cells were secreting antibody fragments, a phenomenon which is clearly shown by the two overlapping distributions. These distributions even stay clearly distinguishable applying high lysozyme concentrations, where a saturation of the fraction of stained cells was reached (Figure 4B). Controls were carried out to check for unspecific binding of first or second detection antibodies which were at least negligible and further reduced by additional wash steps. CLSM was used to illustrate the special labeling of ABF D1.3 scFv producing cells (Figure 4D). Here secreted ABFs were detected by detection antibodies coupled to the fluorochrome Alexa Fluor 488 in the space between cell membrane and cell wall indicated by green ring structures. In addition, Immuno field emission scanning electron microscopy (Immuno-FESEM) was used to detect certain regions of secretion on the cell surface in detail (Figure 4C). Here a second antibody coupled to gold particles was used to make these regions visible. Both methods reveal cells with higher labeling or less labeling giving hints on production and secretion heterogeneities within the bacterial population.

At following investigations of production intensities of *B. megaterium *cells producing ABF D1.3 scFv, two different lysozyme concentrations of first 0.5 mg/L and second 0.025 mg/L were used to check for ABF accumulated at the cytoplasmatic membrane and within the cell wall structures as well. Based on the microscopy pictures evidence, the used detection antibodies can be considered as surface markers and, as such, a quantitative fluorescence surface density (FSD) can be estimated from the raw data of EV and FL1 values.

**Figure 5:**
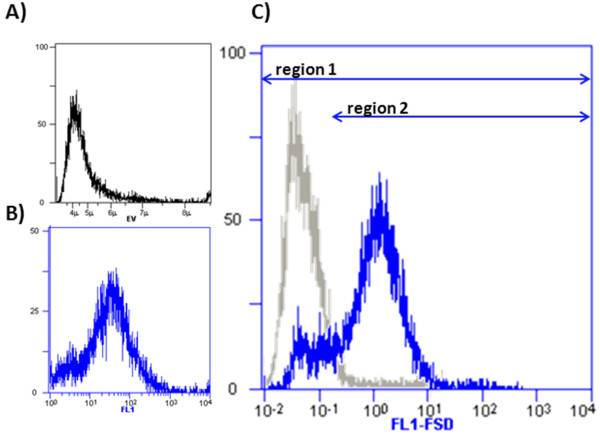
**Definition: Production Intensity**. FL1 Fluorescent Surface Density (FL1-FSD) distributions combined from FL1 and Electronic Volume (EV) data sets. Direct comparison of ABF D1.3 scFv producing (blue) and non- producing cells (grey). Different regions (1, 2) setting to calculate production/secretion intensities of *B. megaterium *cells as shown in formula 1.

The interpretation of estimated FSD is done by differentiated region analysis. As shown in figure 5, two zones were clearly defined. The first region includes all measured cells whereas the second region includes only cells which are labeled with detection antibodies and therefore have an ABF producing status. Taking the ratio of the particular fractions of cells and weightening this with the mean fluorescence value of the second region a quatitative parameter which stands for the production intensity (Prod_Inten) of the whole cell population can be used as bioprocess monitor parameter (equation 1).

### Advanced bioprocess monitoring

The established methods for MP measurements, viability considerations and specific investigation regarding the production status of *B. megaterium *cells producing and secreting ABF D1.3 scFv were applied on samples of a bioreactor cultivation showing the potential of at-line flow cytometric data analysis on single cell level for bioprocess state estimation.

**Figure 6:**
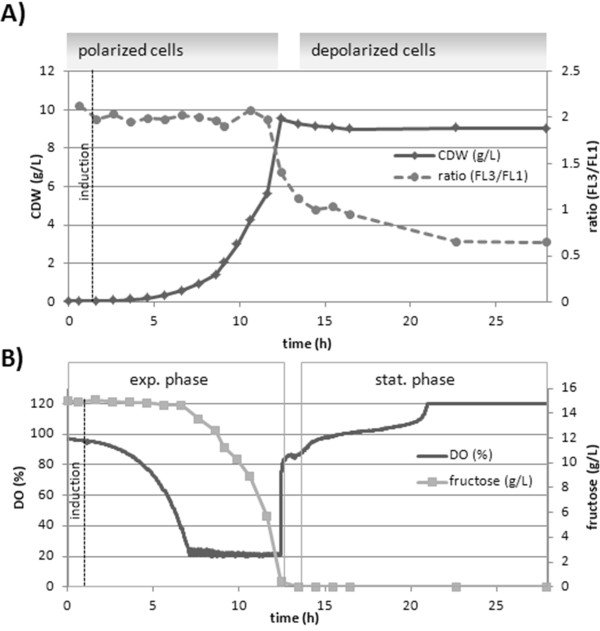
**Bioprocess monitoring: MP estimation**. MP investigations of *B. megaterium *cells producing/secreting ABF scFv D1.3 in a bioreactor cultivation (15 g/L fructose, DO>20%, 1L). **A) **DiOC_2_(3) stain of cells analyzed with ratio analysis of FL3/FL1 values, related to cell dry weight measurements, **B) **Dissolved oxygen (DO), fructose concentration and resulting growth phases.

As shown in figure 6A, B the estimated MP by staining with DiOC_2_(3) could be clearly related to different growth phases of the *B. megaterium *cultivation. Especially during the exponential growth phase the polarization status of the cells was at maximum indicated by the FL3/FL1 ratio analysis shown. As soon as fructose as carbon source was depleted, the dissolved oxygen (DO) concentration immediately increased (Figure 6B) accompanied with a parallel remarkable decrease in FL3/FL1 ratio directly reflecting the reduced MP of the cells. The MP further collapses in the following 10 hours of starvation phase to a constant value corresponding to cells in stationary phase as shown before (Figure 1).

**Figure 7:**
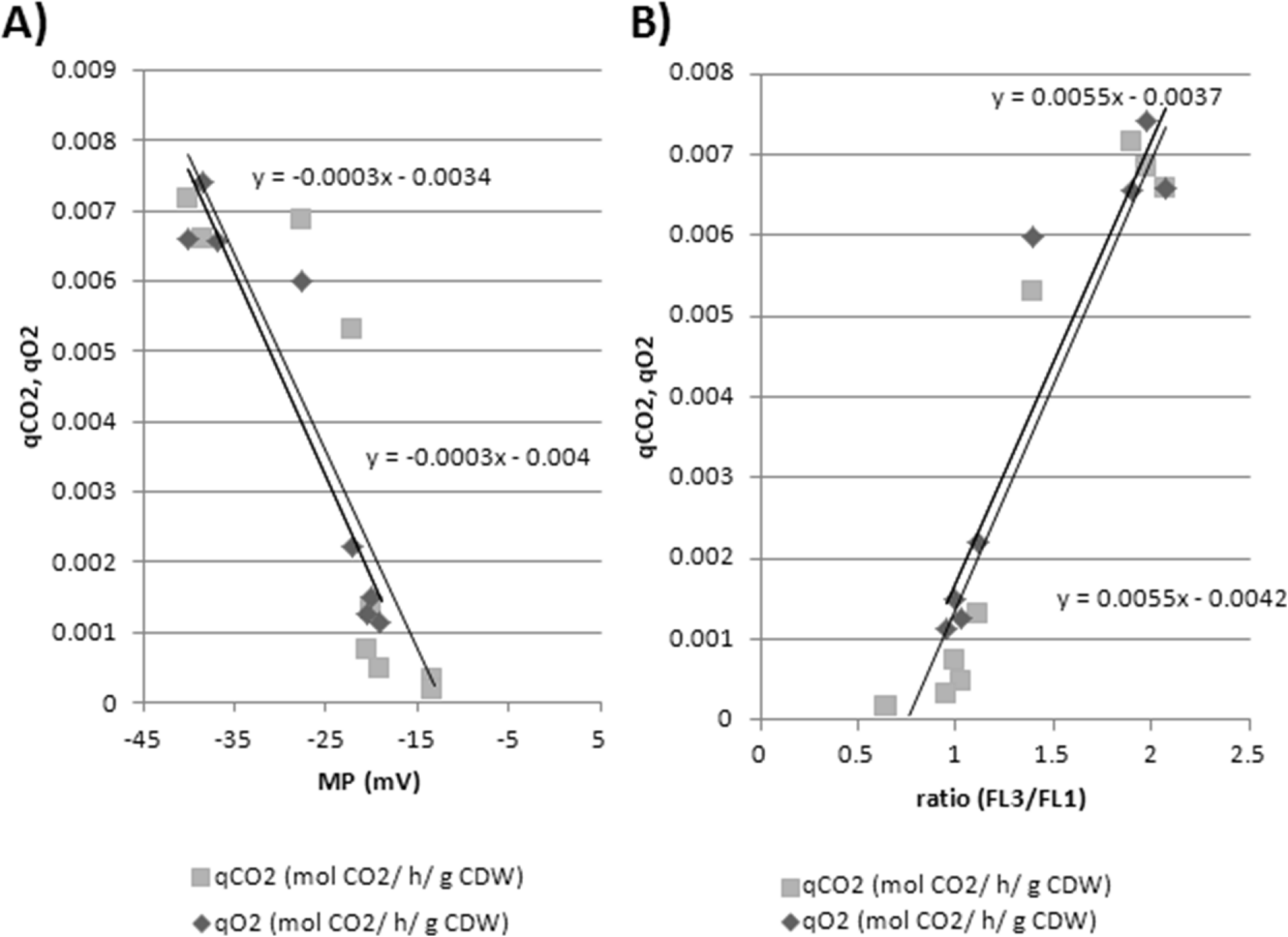
**Bioprocess monitoring: MP-qCO**_**2**_**,qO**_**2 **_**correlation**. Correlation between specific CO_2 _production (qCO_2_), O_2 _consumption rate (qO_2_) with MP measurement deduced from MP calibration and FL3/FL1 ratio analysis.

Interestingly the measured FL3/FL1 ratio values and corresponding MPs (Figure 2) showed a linear correlation with the specific CO_2 _production (qCO_2_) and O_2 _consumption rates (qO_2_) (Figure 7). This illustrates a direct relation between cell MP and microbial metabolic activity represented by CO_2 _production and O_2 _consumption. The correlation holds for any growth phase as the proportionality constant is equal for batch qO_2 _and qCO_2 _variables.

**Figure 8:**
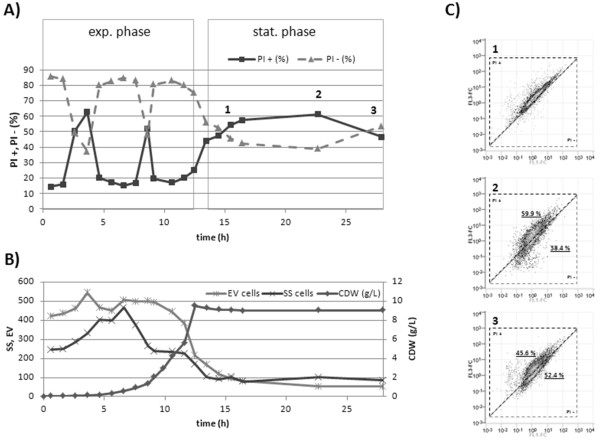
**Bioprocess monitoring: Viability and physiology**. Viability investigations of a bioreactor cultivation of *B. megaterium *cells producing/secreting ABF scFv D1.3 (15 g/L fructose, DO>20%, 1L). **A) **DiBAC_4_(3)/PI stain of samples, percentages of PI+ and PI- cells according gate analysis of FL1-FC and FL3-FC dot plots. **B) **Side Scatter (SS), Electronic Volume (EV) mean value measurements related to cell dry weight characteristics at different growth phases. **C) **Population analysis of cells at late cultivation time points.

The same bioprocess was investigated towards the cell integrity of *B. megaterium *cells. Under these circumstances, only the live/dead test using DiBAC_4_(3)/PI seemed to be applicable or at least sensitive enough to reveal differences in particular cell populations especially in the late stationary phase. It also has to be taken into account that cell characteristics of unstained cells like SS and EV were remarkably affected throughout the cultivation (Figure 8B). Especially the EV was more than halved when the culture developed from exponential to stationary phase. Therefore, normalization by EV values at calculating fluorescent concentration (FL-FC) is very important to interpret fluorescence data sets [30]. Region analysis of dot plots (FL1-FC vs. FL3-FC) was done and percentages of cells in particular regions were determined for all sample points (Figure 8A). Here an increased percentage of cells with higher red fluorescence were partially measured in exponential as well as in stationary phase. These PI positive cells in the exponential phase are probably related to loosen cell wall structures of fast dividing cells [28]. In the stationary phase cell wall structures were reduced and more permeable caused by enhanced autolysin activity [19,31,32].

Finally in the last two sample points a separation into different populations became observable (Figure 8C). Original homogenous populations changed into two fractions and may be related to a population dynamic behavior of long term starvation cultures. Therefore cells from the bioreactor cultivation up to 6 h after reaching the stationary phase should be considered more as dormant or partially damaged (PI permeable) than as totally dead cells although they show an increased PI staining towards long term starvation.

**Figure 9:**
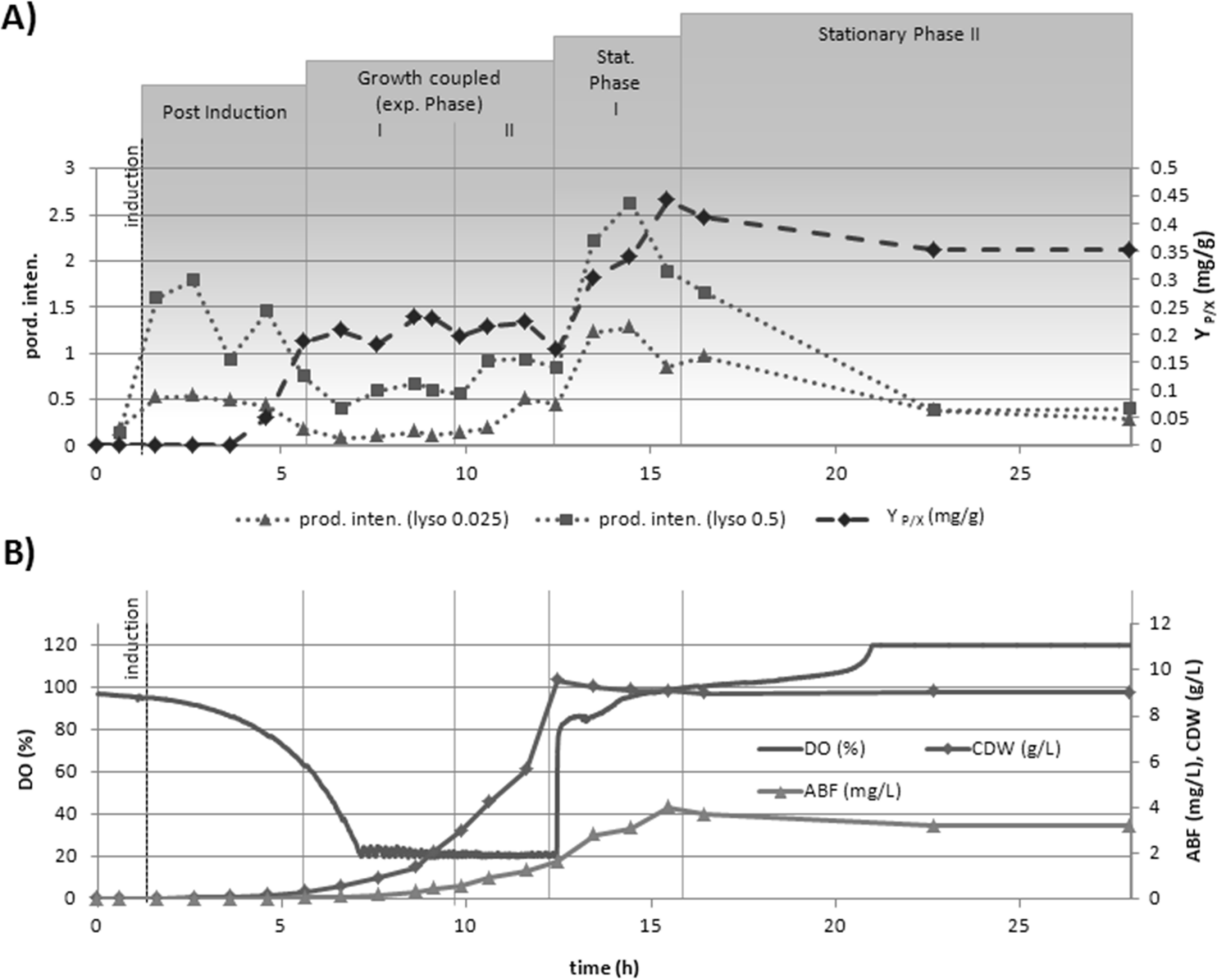
**Bioprocess monitoring: Productivity and Production Intensity**. Product yield (Y_P/X_) and Production Intensity measurements of *B. megaterium *cells producing/secreting ABF scFv D1.3 in a bioreactor cultivation (15 g/L fructose, DO>20%, 1L). **A) **Growth phase dependent product yield (Y_P/X_) and production intensity of ABF D1.3 scFv production and secretion. Production intensities were determined with applying different lysozyme concentrations. **B) **Relation to growth phase dependent dissolved oxygen (DO), absolute ABF scFv D1.3 and dry biomass concentration.

The underlying bioprocess was also checked for production of ABF D1.3 scFv regarding the efficiency of secretion of single cells and over all yield considerations. Therefore the previously defined production intensity was determined via fluorescence labeling and the functionally secreted and folded ABF D1.3 scFv concentrations were measured in the supernatant by ELISA assay. These absolute concentrations of ABF were normalized by the measured cell dry weight (yield coefficient (Y _P/X_)) and both calculated parameters "production intensity" and "Y _P/X_" were directly compared (Figure 9). To get an idea of secretion efficiency of ABF through the cell wall, different lysozyme concentrations were applied after cell fixation. By using a high concentration of lysozyme it was assumed to directly reflect the amount of ABF accumulated behind the cell membrane. At low concentrations of lysozyme, ABF within the cell wall structures might be detected due to partially degradation of cell wall components.

In the investigated batch process, different phases of production could be clearly distinguished. The first phase right after induction showed a remarkably increase in the production intensity at both 0.025 g/L and 0.5 g/L lysozyme treatment (Figure 9). The product yield remains low and has carefully to be interpreted due to low concentrations of biomass and secreted ABF concentrations in the detection limit. At exponential growth both the production intensity as well as the product yield remain constant which is related to growth coupled production behavior. Here the increasing of biomass directly favors higher concentrations of functional folded ABF in the supernatant in the same range (Figure 9B). A detailed view reveals higher production intensities in the late phase of exponential growth. Of particular importance is the time shift observed in this phenomenon for the different lysozyme treatments. At lower lysozyme concentrations the shift to higher production intensities is 1 h later compared to lysozyme treatment with higher concentration. This may be explained by a previous accumulation of ABF behind the membrane followed by at least 1 h lasting release through the wall. This delay of the secretion also becomes obvious in the stationary phase. Here a sudden increase in production intensity of both lysozyme treatment approaches is directly followed by a delayed increase in the production yield. A definite decrease in production intensity estimated through single cell analysis was shown in the late stationary phase, which is probably related to starvation and cell inactivation. However the antibody fragment concentration as well as the biomass concentration remains constant.

As both time courses of the product yield Y _P/X _and the production intensity are coupled, it can be concluded that the secretion process of ABF and the product biosynthesis process inside the cell is directly related to the final secretion and functional appearance outside the cell in the supernatant. Interestingly the measured production intensities of particular lysozyme treatments show more distinctive differences at phases of higher production. This stresses that the cell wall itself is a barrier which has to be overcome by secreted ABF. Therefore, especially at states of high production, the cell wall may be regarded as a bottleneck concerning the observed overall process productivity, since it delays the product transport increasing the secretion time at single cell level.

Through analysis of MP and production intensities no population heterogeneities within the different growth and production phases became observable. This is most probably due to well controlled bioprocess with no oxygen limitation, temperature and pH-control with ideal mixing conditions.

## Discussion

Single cell analysis of *B. megaterium *cells producing and secreting ABF was shown to be feasible in giving detailed insights and better understanding of the underlying bioprocess. Established viability and production intensity assays revealed different cell states of active, non-active, dormant, producing, non-producing and dead cells.

It should be stressed that protocols and assays have to be basically developed for the particular organisms which are in the current focus of investigation. For MP estimation different dyes were evaluated in shaking flask experiments and tested in a bioreactor cultivation. Although DiBAC_4_(3) and DiOC_6_(3) were capable to measure MP in calibration experiments they were found not to be effective for physiological cell characterization at bioreactor cultivations. This may be due to changes in cell properties related to cell wall and membrane structures caused by an increased shear stress in bioreactors compared to shaking flask experiments. Cell wall and membrane structures may influence the characteristics of dye binding [27]. However, DiOC_2_(3) was found to be most applicable in describing the status of cell membrane polarization under bioreactor culture conditions as well and, in contrast to other dyes, caused less changes in cell properties themselves (Table 1). This better applicability may be related to the higher concentrations used for DiOC_2_(3) based on saturation and agglomeration at polarized membranes [27] probably resulting in a more robust assay.

Evaluation of cell viability was done both with DiBAC_4_(3)/PI and Syto9/PI combinations, but only DiBAC_4_(3)/PI was sensitive enough to reveal differences and even population dynamics at late stages of starvation of the bioreactor cultivation. When using PI as an indicator for cell integrity it has to be taken into account, that it not only stains dead cells but may label highly reproductive cells as well [28] and probably due to its quenching properties, PI reduces the Syto 9 and DiBAC_4_(3) green fluorescence intensity. Significant variability in uptake patterns of nucleic acid binding dyes by Gram positive bacteria was shown before [33].

Comparing both at line methods for cell status evaluation, it becomes clearly obvious, that MP measurements by DiOC_2_(3) are by far more sensitive in describing cell properties than the presented assays for cell integrity measurements. The latter are more accurate in measuring extreme effects like cell lysis after heat treatment or long term starvation. As both parameters of polarization status and viability are important, they should be monitored simultaneously at bioreactor cultivations and are most helpful in describing population dynamics and cell physiology. Especially in deciding whether a process is operating in a stable condition or not, the MP estimation is an ideal sensitive parameter to adapt process conditions or even abort the cultivation at early stage when heterogeneities become present or critical physiological values are reached. In the area of omics techniques and fast sampling, the insurance that a homogeneous population is characterized is also a very important issue [34] which can be quantitatively analyzed by at line MP measurements. The implementation of cytomics and proteomics technique regarding bacterial cells was recently proven to be successful [35].

Throughout the whole bioprocess it became clear that cell properties like SS, representing the granularity of cells, and the electronic volume (cell size) are changing during the cultivation (Figure 8). This has to be taken into account and probably has particular distinctive effects on staining intensity and behavior. Physiological state changes in cell size and granularity e.g. protein or PHB content have been shown to be dependent on growth phases for other bacteria before [12,36-39]. In addition, a growth phase dependent behavior of *B. megaterium *cell size can arise from cell chain formation. In case of chain formation, it is not possible to know if these chains are further separated into single cells through the hydrodynamical focusing of the flow cytometer. In the device used here, the equivalent size and fluorescence are estimated for very "event", i.e. for every counted particle no matter if this is a single bacterium or a chain of bacteria. Normalizing fluorescence to cell size is done per event, so that the normalization actually represents fluorescence per cell or cell conglomerate no matter how many (single or chains) or how big these cells (single or chains) are [30].

It was shown, that the developed method for discriminating between producing and non-producing cells was feasible to characterize the production status bioreactor cultivations and gives not only qualitative, but also quantitative estimations of production intensities. This differentiation is the main pre-condition for population analysis under limiting process conditions and high cell density cultivations, where heterogeneities are more likely to occur.

The used Alexa Dye 488 is a bright photostable conjugate being most applicable in measuring reproducible and reliable results with a high resolution [40]. The established method of determining production intensities of single cells also revealed certain dynamics within the process of secretion through the membrane coupled SEC pathway [41] and subsequent release from the cell wall structures. Here a certain delay in secretion became remarkably obvious. To determine secretion rates of yeast cells other methods relying on artificially created affinity matrices have been developed before [42,43]. In this case, the developed method is more simple and robust, based on fixation and lysozyme treatment of *B. megaterium *cells. Although cells have to be considered as dead after paraformaldehyde treatment they could be sorted and are suitable for transcriptomic or proteomic analyses [35,44]. Even plasmid banks of *B. megaterium *could be used for screening for high producers similar to library screens of scFv libraries [45,46].

Cell integrity, MP and production intensity measurements revealed clear changes in the particular cell status at the transition from exponential to stationary phase. Here the question arises if the depolarization of cells in the stationary phase accompanied with decreased membrane integrity indicated by slightly increased PI staining may be directly related to the gain in production and secretion of antibody fragment D1.3 scFv. It was shown before that decreasing the polarization status of a cell may be directly linked to a better secretion performance of proteins due to changes in the cell structure based on higher autolysin activity, which depends on the cell wall charge distribution [32]. Therefore MP estimation may be a related parameter to production and secretion of ABF D1.3 scFv and therefore could be used for further process control.

By the analysis done it was discarded that cells in the early stationary phase 2-3h after fructose depletion should be considered as dead and, therefore, lyse and release ABF in a high amount. Moreover, entirely depolarized cells in a dormant status are totally recovered by fructose addition after starving periods of up to 4 hours (results not shown). Nevertheless measured FL3-FC values of PI treated samples should be treated very carefully. A relatively high red fluorescence of cells in the exponential phase may be related for instance to porose cell wall or cell membrane structure of fast dividing cells [28].

## Conclusions

Microorganisms in industrial processes are considered conventionally as uniform populations and therefore are thought to be sufficiently described by average values. Especially for high cell densities reached at fed batch processes it was shown that this is not true [13,47-49]. With *E. coli *cultivations even a reduction of up to 20% of cell viability was observed during the fed-batch processes [50]. Therefore it's most desirable to have reliable methods available to adequately monitor bioprocesses for production intensities of single cells and reveal population dynamics, e.g. under substrate limited conditions or high cell densities. Particularly in large scale reactors heterogeneities are more common due to concentration gradients of oxygen or substrate [51]. Here, the estimation of heterogeneities would directly reflect the quality of a bioprocess or even enable special reactor design and/or particular feeding strategies.

Further application of flow cytometry in the future may be found in online measurements and even online control of bioprocesses as it was shown to be applicable in fed batch processes for mammalian cells [52]. In combination with appropriate models for population dynamics, flow cytometry may be realized to establish an online optimization process to most effective, robust and productive bioprocesses.

## Methods

### Cultivation conditions

All chemicals were purchased from Sigma (Steinheim, Germany), Merck (Darmstadt, Germany) or Roth (Karlsruhe, Germany) and are of analytical grade. In all experiments the *B. megaterium *strain YYBm1 [16] carrying the plasmid pEJBmD1.3 scFv was used [19]. For the preculture and main culture of *B. megaterium *a minimal medium was applied [23]. It contains fructose as a sole carbon source supplemented with 3.52 g L^-1 ^KH_2_PO_4_, 5.297 g L^-1 ^Na_2_HPO_4_, 3 g L^-1 ^MgSO_4 _× 7 H_2 _O, 25 g L^-1 ^(NH_4_)_2_SO_4_, 0.312 g L^-1 ^MnCl_2 _× 4 H_2_O, 0.0954 g L^-1 ^CaCl_2 _× 2 H_2_O, 5.5 mg L^-1 ^FeSO_4 _× 7 H_2_O, 21.6 mg L^-1 ^(NH_4_)_6_Mo_7_O_24 _× 4 H_2_O, 6.2 mg L^-1 ^CoCl_2_, 16 μg L^-1 ^CuSO_4 _× 5H_2_O, 155 μg L^-1 ^H_3_BO_3_, 15 μg L^-1 ^ZnSO_4 _× 7H_2_O and 10 mg L^-1 ^tetracycline. For the pre-culture and shaking flask experiments the concentration of fructose was 5 g L^-1 ^and for the bioreactor cultivation 15 g L^-1^, respectively.

All cultivations in shaking flasks were done at 130 rpm and 37°C (50 mm shaking diameter, CERTOMAT^® ^BS-1, B. Braun Biotech International, Melsungen, Germany). *B. megaterium *was stored at -80°C in a 50% glycerol solution in cryo vials. For preparing cryo cultures *B. megaterium *was cultivated in 10 mL minimal medium at 37°C for 12 h. 100 mL minimal medium in a 1000 mL shaking flask without baffles were inoculated with this cell suspension adjusting to an OD_578 nm _of 0.1. In the exponential phase (OD_578 nm _= 3) 10 mL of cell suspension were mixed with 10 mL glycerol (99%) and immediately frozen in 0.5 ml aliquots in liquid nitrogen and stored at -80°C. For pre-cultures, 100 μL of cryo culture were used as inoculum for 10 mL of minimal medium and cultivated for 12 hours. As second pre-culture for bioreactor experiments, 100 mL medium was inoculated from first pre-culture adjusting to an OD_578 nm _of 0.1 and cultivated for additional 12 hours. Main cultivation was carried out in a 3.7 L bioreactor (Bioengineering, Wald, Switzerland). For the batch experiment 1 L of minimal medium was inoculated with a pre-culture volume adjusting to an OD_578 nm _of 0.1. The cultivation was carried out under constant temperature at 37°C and controlled at pH 6.3. Dissolved oxygen (DO) concentration was controlled above a value of 20% saturation, using a stepwise cascade control, increasing alternately agitation and aeration rate. Induction was done by an appropriate amount of a 100 g L^-1 ^xylose solution 1.5 hours after inoculation to reach a final concentration of 5 g L^-1^.

### Gas analysis

Carbon dioxide and oxygen in the exhaust gas were measured with gas sensors (Blue Sense, Herten, Germany) and the corresponding specific oxygen uptake rate (qO_2_) and specific carbon dioxide production rate (qCO_2_) were calculated for all cultivations considering nitrogen as inert gas.

### Sampling of cultivation supernatant

Samples were taken for biomass, xylose, fructose concentration measurements and single cell analysis. Cell suspension (2 mL) was centrifuged at 15.7 g, 5 min, 4°C (5415 R Eppendorf, Hamburg, Germany). For MP measurement the centrifugation was done at 3.3 g and cells were immediately stained and measured after taking the sample. Supernatant was frozen at -20°C and cell pellet was stored at 4°C for production intensity assay. Supernatant was used for determining sugar concentration by HPLC analysis and for quantitative ELISA test as described below. Cell pellets were used for determining production intensities, cell dry weight concentration (CDW) and Electron Microscopy (EM).

### Analytics of substrates, products and biomass

Sugars (xylose, fructose) were quantified by HPLC (Hitachi Elite LaChrom, Krefeld, Germany) equipped with a Metacarb 87 C column (Varian, Palo Alto, CA, USA) as stationary phase and Millipore H_2_O as mobile phase at 0.6 mL min^-1 ^and 85°C. Detection was performed using an IR detector.

For preparation of samples 2 mL of the cultivation supernatant was sucked into a 2 mL plastic syringe and directly squeezed through a sterile filter (polyvinylidene fluoride, 0.2 μm pore size, Roth, Karlsruhe, Germany). For protein precipitation 20 μL mL^-1 ^0.5 M H_2_SO_4 _were added and samples were frozen at -20°C. After thawing and centrifuging at 15.7 g, 4°C, 5 min (5415 R Eppendorf, Hamburg, Germany) 500 μL of supernatant was filled in HPLC vials and analyzed.

Cell concentration was determined as optical density at a wavelength of 578 nm using a Novespec 3 Photometer (Amersham Bioscience, Freiburg, Germany). CDW was measured via gravimetric analysis. Here biomass pellets of 15 mL culture volume (triplicats) were washed twice with distilled water to remove salts and afterwards dried at 80°C for 48h.

### ELISA

ELISA was carried out according to Jordan et al. (2007). A quantitative determination of functional folded antibody fragment D1.3 scFv was done by measuring a calibration standard of purified ABF D1.3 scFv on each 96 well plate. The standard was purified from supernatant with a protein-L (©Pierce) column and quantified by densiotometric analysis of corresponding SDS gel bands related to a D1.3 scFv standard. Samples were measured in three dilutions (1:3) and quantified according to the standard samples via logit-log plot analysis.

### Production intensity calculation

The production intensity was calculated according to single cell measurements of Alexa Fluor 488 labeled cells based on region analysis (Figure 5) and the formula below:(1)

### Flow cytometry

Flow cytometry studies were performed using a Quanta MPL (Beckmann Coulter, Krefeld, Germany) flow cytometer equipped with a 488 nm argon Laser. The SS signal was used as a trigger signal, green fluorescence (FL1) was detected through a dual long pass filter (525 nm, +-21 nm bandwidth) and red fluorescence (FL3) was detected through a 670 nm long pass filter. Both fluorescence values were detected in parallel using a dichroic long pass filter for splitting. Sheath flow rate was 4.17 μl min^-1^, sample rate never exceeded over 600 events s^-1 ^and 10,000 counts were done. Signals were logarithmically amplified and photomultiplier (PMT) settings were adjusted to particular staining methods. The EV (Coulter Counter Principle, Cell Lab QuantaTM SC MPL) was used to determine cell fluorescence concentration (FL-FC: FL-Channel/(Volume Channel)) and cell fluorescence surface density (FL-FSD: FL-Channel/((Volume Channel) ^ (2/3)).

### Dye screening for MP estimation

The different dyes DiOC_2_(3), DiOC_6_(3) and DiBAC_4 _(3) were tested according their suitability for MP estimation. Therefore 1 mL of *B. megaterium *cells of particular samples with a concentration of 2 × 10^6 ^cells mL^-1 ^were centrifuged (3.3 g, room temperature) and resuspended in 1 mL staining puffer (0.06 M Na_2_HPO_4_, 0.06 M NaH_2_PO_4_, 5 mM KCl, 130 mM NaCl, 1.3 mM CaCl_2_, 0.5 mM MgCl_2 _adjusted to a pH 7 with NaOH, steril filtered). 10 μl of particular dye working solution was added (DiOC_2_(3), DiOC_6_(3) in 50%/50% -DMSO/H_2_O, DiBAC_4_(3) in 100% DMSO). As a negative control 10 μl CCCP solution (1.5 mM in DMSO) was added to depolarize cells. All dyes and CCCP were purchased from Invitrogen (Molecular Probes, USA).

### DiOC_2_(3) ratio analysis

Ratio analysis of DiOC_2_(3) stain was done according Novo et al. 1999 and was used to estimate MP.(2)

Polarized cells show an increased staining property with the actually green fluorescent dye leading to a dye accumulation with red fluorescent properties [29].

### Membrane potential calibration

MPs were artificially simulated by the application of the potassium ionophore valinomycin in the presence of different external potassium concentrations [25]. At particular potassium concentrations, the resulting FL3/FL1 ratios of DiOC_2_(3) stained cells were determined. Further MP were calculated based on the Nernst Equation (ΔE= - 61.54 mV log (c_inside_/c_outside_)) where potassium concentration inside the cells was assumed to be constant at 243.75 mM. Potassium concentrations outside the cells were changing according to experimental setup from 300 mM to 0 mM where the overall molarity was kept constant at 300 mM by adding an appropriate amount of sodium. Staining was done in buffer of 0.06 M Na_2_HPO_4_, 0.06 M NaH_2_PO_4_, 130 mM NaCl, 1.3 mM CaCl_2 _and 0.5 mM MgCl_2 _adjusted to a pH 7 with NaOH, steril filtered and addition of the appropriate amount of NaCl and KCl.

### Single cell production intensity assay

After sampling a cell pellet of about 10^7 ^cells, cells were re-suspended in 200 μL PBS-T (PBS (8.5 g L^-1 ^NaCl, 1.34 g L^-1 ^Na_2_HPO_4 _× 2H_2_O, 0.345 g L^-1 ^NaH_2_PO_4 _× 2H_2_O), 0.05% Tween 20) and 600 μL of 4% paraformaldehyd was added. Cells were fixed for 10 minutes at room temperature. After centrifugation at 15.7 g for 5 min at 4°C the cell pellet was resuspended in 900 μL PBS-T and 100 μL of particular lysozyme stock solution (0.25 mg mL^-1 ^to 5 mg mL^-1 ^in PBS-T) was added. After incubation for 10 min at room temperature and centrifugation at 15.7 g, 5 min, 4°C cells were washed once with 500 μL PBS-T to reduce unspecific binding. Cells were incubated with first detection antibody mouse-anti-penta-His (100 μL, 1:100 in PBS-T, Quiagen 34660) at RT for 1.5 h and afterwards washed twice with 500 μL PBS-T. Second Alexa Fluor coupled antibody (100 μL, 1:50, Alexa Fluor 488 ^® ^goat anti-mouse IgG (H+L) highly cross-adsorbed 2 mg mL^-1 ^Cat.No. A-11029 Invitrogen, USA) was added and incubated for 1 h at 4°C in the dark. Cells were washed once with 500 μL PBS-T and flowcytometric analysis was performed using the green fluorescence channel. Negative controls were established for cells treated with no lysozyme, not induced cells and leaving of first or second detection antibody.

### Viability assay

For distinguishing between live and dead cells two viability assays were evaluated. Here Syto9/PI (LIVE/DEAD Baclight Kit, Invitrogen, USA) and DiBAC_4_(3)/PI were used for different mixtures of live (exp. phase) and heat treated cells (80°C, 10 min). As described before, 10 μL of particular staining working solutions were added to 2 × 10^6 ^cells in 1 mL staining puffer (PI: 1.5 mM, Syto9: 500 μM, DiBAC_4_(3): 0.21 mM). Staining patterns in green and red fluorescence were subsequently analyzed by flow cytometry.

### Immuno field emission scanning electron microscopy (Immuno-FESEM)

Preparing samples for electron microscopy cells were treated as described before under "single cell productions intensity assay" with the addition that as a second detection antibody 15 nm goat antimouse-IgG antibodies coupled to 15 nm gold nanoparticles were applied. After washing with PBS samples were adsorbed onto butvar-coated 300 mesh copper grids, washed with TE buffer (20 mM TRIS, 1 mM EDTA, pH 6,9), distilled water and air-dried. Samples were then mounted onto conductive carbon adhesive tabs on specimen mounts. Samples were examined in a Zeiss DSM982 Gemini field emission scanning electron microscope (Carl Zeiss, Germany) at an acceleration voltage of 5 kV using the Everhart-Thornley secondary electron detector (SE-detector) and the built-in inlens Se-detector in a 75:25 ratio. Images were recorded onto MO-disk and contrast and brightness was adjusted with Adobe Photoshop CS4.

### Confocal laser scanning microscopy (CLSM)

The CLSM technique was applied to directly monitor bound Alexa Fluor detection antibodies at the cell surface. Here cells were resuspended in PBS and investigated with CLSM technique. Fluorescence was analyzed with a confocal laser scanning microscope CLSM-510META connected to an Axiovert 200 M (Carl Zeiss, Germany) with laser excitation of 488 nm, HFT UV/488 and BP 505-530 for GFP fluorescence. All images were processed with LSM Image Browser Release 4.2 (Carl Zeiss, Germany).

## Abbreviations

ΔE: Membrane Potential (Nernst Equation); AB: antibody; ABF: Antibody fragment; ATP: Adenosine-5'-triphosphate; CCCP: carbonyl cyanide m-chlorophenylhydrazone; CDW: Cell Dry Weight; c_inside_: potassium concentration inside the cells; CLSM: Confocal Laser Scanning Microscopy; c_outside: _potassium concentration outside the cells; DFG: Deutsche Forschungsgemeinschaft; DiBAC_4_(3): Bis - (1,3 - dibutylbarbituric acid) trimethine oxonol; DiOC_2_(3): 3,3' - Diethyloxacarbocyanine iodide; DiOC_6_: (3,3'-dihexyloxacarbocyanine iodide); DMSO: Dimethyl sulfoxid; DO: dissolved oxygen; ELISA: Enzyme-linked immunosorbent assay; EV: Electronic Volume; FC: Fluorescence Concentration; FESEM: Field Emission Scanning Electron Microscopy; FDA: Food and Drug Administration; FL1: Fluorescence 1 (Green); FL3: Fluorescence 3 (Red); FSD: Fluorescence Surface Density; HPLC: High Performance Liquid Chromatography; HZI: Helmholz Zentrum für Infektionsforschung; IMAC: Immuno Metall Affinity Chromatography; MP: Membrane Potential; NADH_2_: Nicotinamide Adenine Dinucleotide; OD: Optical Density; PAT: Process Analytical Technologies; PBS: Phosphate Buffered Saline; PBS-T: Phosphate Buffered Saline Tween; PFA: Paraformaldehyde; PHB: Polyhydroxybutyrat; PI: Propidium Iodide; PMT: Photomultiplier; Prod_Inten: Production Intensity; qCO_2_: specific CO_2 _production rate; qO_2_: specific O_2 _consumption rate; scFv: single-chain variable fragment; SEC: secretion; SS: Side Scatter;

## Competing interests

The authors declare that they have no competing interests.

## Authors' contributions

FD designed the study and experiments. Experiments were carried out and discussed by FD and AB. EFL coordinated the study, discussed data and helped to draft the manuscript. All authors read and approved the final manuscript. RH did the CLSM pictures and MR took the Immuno-FESEM images.

## References

[B1] Müller S Harms H Bley T Origin and analysis of microbial population heterogeneity in bioprocesses Current opinion in biotechnology 2010 21 100 113 20138500 10.1016/j.copbio.2010.01.002

[B2] Reis A da Silva TL Kent CA Kosseva M Roseiro JC Hewitt CJ Monitoring population dynamics of the thermophilic Bacillus licheniformis CCMI 1034 in batch and continuous cultures using multi-parameter flow cytometry Journal of biotechnology 2005 115 199 210 10.1016/j.jbiotec.2004.08.005 15607238

[B3] Walberg M Gaustad P Steen HB Rapid flow cytometric assessment of mecillinam and ampicillin bacterial susceptibility Journal of Antimicrobial Chemotherapy 1996 37 1063 10.1093/jac/37.6.1063 8836810

[B4] Gauthier C St-Pierre Y Villemur R Rapid antimicrobial susceptibility testing of urinary tract isolates and samples by flow cytometry Journal of medical microbiology 2002 51 192 11871613 10.1099/0022-1317-51-3-192

[B5] Suller MTE Lloyd D Fluorescence monitoring of antibiotic-induced bacterial damage using flow cytometry Cytometry Part A 1999 35 235 241 10.1002/(SICI)1097-0320(19990301)35:3<235::AID-CYTO6>3.0.CO;2-0 10082304

[B6] Bunthof CJ Bloemen K Breeuwer P Rombouts FM Abee T Flow cytometric assessment of viability of lactic acid bacteria Applied and environmental microbiology 2001 67 2326 10.1128/AEM.67.5.2326-2335.2001 11319119 PMC92874

[B7] Rault A Béal C Ghorbal S Ogier JC Bouix M Multiparametric flow cytometry allows rapid assessment and comparison of lactic acid bacteria viability after freezing and during frozen storage Cryobiology 2007 55 35 43 10.1016/j.cryobiol.2007.04.005 17577587

[B8] Boyd AR Gunasekera TS Attfield PV Simic K Vincent SF Veal DA A flow-cytometric method for determination of yeast viability and cell number in a brewery FEMS yeast research 2003 3 11 16 12702241 10.1111/j.1567-1364.2003.tb00133.x

[B9] Czechowska K Johnson DR van der Meer JR Use of flow cytometric methods for single-cell analysis in environmental microbiology Current opinion in microbiology 2008 11 205 212 10.1016/j.mib.2008.04.006 18562243

[B10] Hewitt CJ Nebe-Von-Caron G An industrial application of multiparameter flow cytometry: assessment of cell physiological state and its application to the study of microbial fermentations Cytometry Part A 2001 44 179 187 10.1002/1097-0320(20010701)44:3<179::AID-CYTO1110>3.0.CO;2-D 11429768

[B11] Gernaey KV Woodley JM Sin G Introducing mechanistic models in Process Analytical Technology education Biotechnology Journal 2009 4 593 599 10.1002/biot.200800323 19396924

[B12] Steen HB Boye E Skarstad K Bloom B Godal T Mustafa S Applications of flow cytometry on bacteria: cell cycle kinetics, drug effects, and quantitation of antibody binding Cytometry Part A 1982 2 249 257 10.1002/cyto.990020409 7035105

[B13] Looser V Hammes F Keller M Berney M Kovar K Egli T Flow-cytometric detection of changes in the physiological state of E. coli expressing a heterologous membrane protein during carbon-limited fedbatch cultivation Biotechnology and bioengineering 2005 92 69 78 10.1002/bit.20575 16142799

[B14] Nebe-von Caron G Stephens B Assessment of bacterial viability status by flow cytometry and single cell sorting Journal of applied microbiology 1998 84 988 998 10.1046/j.1365-2672.1998.00436.x 9717283

[B15] Vary PS Plasmidless strain of Bacillus megaterium QM B1551 1991 Google Patents

[B16] Yang Y Biedendieck R Wang W Gamer M Malten M Jahn D Deckwer WD High yield recombinant penicillin G amidase production and export into the growth medium using Bacillus megaterium Microb Cell Fact 2006 5 36 10.1186/1475-2859-5-36 17132166 PMC1687198

[B17] Malten M Biedendieck R Gamer M Drews AC Stammen S Buchholz K Dijkhuizen L Jahn D A Bacillus megaterium plasmid system for the production, export, and one-step purification of affinity-tagged heterologous levansucrase from growth medium Applied and environmental microbiology 2006 72 1677 10.1128/AEM.72.2.1677-1679.2006 16461726 PMC1392972

[B18] Furch T Wittmann C Wang W Franco-Lara E Jahn D Deckwer WD Effect of different carbon sources on central metabolic fluxes and the recombinant production of a hydrolase from Thermobifida fusca in Bacillus megaterium J Biotechnol 2007 132 385 394 10.1016/j.jbiotec.2007.08.004 17826861

[B19] Jordan E Hust M Roth A Biedendieck R Schirrmann T Jahn D Dübel S Production of recombinant antibody fragments in Bacillus megaterium Microbial Cell Factories 2007 6 2 10.1186/1475-2859-6-2 17224052 PMC1797049

[B20] Dübel S Handbook of therapeutic antibodies 2007 Wiley-VCH

[B21] Pelat T Hust M Hale M Lefranc M Dübel S Thullier P Isolation of a human-like antibody fragment (scFv) that neutralizes ricin biological activity BMC Biotechnology 2009 9 10.1186/1472-6750-9-60 19563687 PMC2716335

[B22] Pelat T Hust M Laffly E Condemine F Bottex C Vidal D Lefranc MP Dubel S Thullier P High-affinity, human antibody-like antibody fragment (single-chain variable fragment) neutralizing the lethal factor (LF) of Bacillus anthracis by inhibiting protective antigen-LF complex formation Antimicrobial agents and chemotherapy 2007 51 2758 10.1128/AAC.01528-06 17517846 PMC1932538

[B23] David F Westphal R Bunk B Jahn D Franco-Lara E Optimization of antibody fragment production in Bacillus megaterium: the role of metal ions on protein secretion Journal of Biotechnology 2010 150 115 124 10.1016/j.jbiotec.2010.07.023 20670661

[B24] Lopes da Silva T Piekova L Mileu J Roseiro JC A comparative study using the dual staining flow cytometric protocol applied to Lactobacillus rhamnosus and Bacillus licheniformis batch cultures Enzyme and Microbial Technology 2009 45 134 138 10.1016/j.enzmictec.2009.03.001

[B25] Novo D Perlmutter NG Hunt RH Shapiro HM Accurate flow cytometric membrane potential measurement in bacteria using diethyloxacarbocyanine and a ratiometric technique Cytometry Part A 1999 35 55 63 10.1002/(SICI)1097-0320(19990101)35:1<55::AID-CYTO8>3.0.CO;2-2 10554181

[B26] Hewitt CJ Onyeaka H Lewis G Taylor IW Nienow AW A comparison of high cell density fed batch fermentations involving both induced and non induced recombinant Escherichia coli under well mixed small scale and simulated poorly mixed large scale conditions Biotechnology and bioengineering 2007 96 495 505 10.1002/bit.21127 16902956

[B27] Sträuber H Müller S Viability states of bacteria-Specific mechanisms of selected probes Cytometry Part A 2010 77 623 634 10.1002/cyto.a.20920 20583280

[B28] Shi L Günther S Hübschmann T Wick LY Harms H Müller S Limits of propidium iodide as a cell viability indicator for environmental bacteria Cytometry Part A 2007 71 592 598 10.1002/cyto.a.20402 17421025

[B29] Shapiro HM Membrane potential estimation by flow cytometry Methods 2000 21 271 279 10.1006/meth.2000.1007 10873481

[B30] Lüders S David F Steinwand M Jordan E Hust M Dübel S Franco-Lara E Influence of the hydromechanical stress and temperature on growth and antibody fragment production with Bacillus megaterium Applied microbiology and biotechnology 2011 10.1007/s00253-011-3193-7 21479717

[B31] Jolliffe LK Doyle RJ Streips UN The energized membrane and cellular autolysis in Bacillus subtilis Cell 1981 25 753 763 10.1016/0092-8674(81)90183-5 6793239

[B32] Kemper MA Urrutia MM Beveridge TJ Koch AL Doyle RJ Proton motive force may regulate cell wall-associated enzymes of Bacillus subtilis Journal of bacteriology 1993 175 5690 8396121 10.1128/jb.175.17.5690-5696.1993 PMC206628

[B33] Walberg M Gaustad P Steen HB Uptake kinetics of nucleic acid targeting dyes inS. aureus, E. faecalis andB. cereus: a flow cytometric study Journal of microbiological methods 1999 35 167 176 10.1016/S0167-7012(98)00118-3 10192050

[B34] Schädel F Franco-Lara E Rapid sampling devices for metabolic engineering applications Applied microbiology and biotechnology 2009 83 199 208 19352648 10.1007/s00253-009-1976-x

[B35] Jehmlich N Hübschmann T Gesell Salazar M Völker U Benndorf D Müller S von Bergen M Schmidt F Advanced tool for characterization of microbial cultures by combining cytomics and proteomics Applied microbiology and biotechnology 2010 1 10 10.1007/s00253-010-2753-6 20676634

[B36] Steen HB Boye E Escherichia coli growth studied by dual-parameter flow cytophotometry Journal of Bacteriology 1981 145 1091 7007339 10.1128/jb.145.2.1091-1094.1981 PMC217223

[B37] James BW Mauchline WS Dennis PJ Keevil CW Wait R Poly-3-hydroxybutyrate in Legionella pneumophila, an energy source for survival in low-nutrient environments Applied and environmental microbiology 1999 65 822 9925622 10.1128/aem.65.2.822-827.1999 PMC91101

[B38] Ackermann J Müller S Lösche A Bley T Methylobacterium rhodesianum cells tend to double the DNA content under growth limitations and accumulate PHB Journal of Biotechnology 1995 39 9 20 10.1016/0168-1656(94)00138-3

[B39] Maskow T Müller S Lösche A Harms H Kemp R Control of continuous polyhydroxybutyrate synthesis using calorimetry and flow cytometry Biotechnology and bioengineering 2006 93 541 552 10.1002/bit.20743 16245347

[B40] Panchuk-Voloshina N Haugland RP Bishop-Stewart J Bhalgat MK Millard PJ Mao F Leung WY Haugland RP Alexa dyes, a series of new fluorescent dyes that yield exceptionally bright, photostable conjugates Journal of Histochemistry and Cytochemistry 1999 47 1179 10.1177/002215549904700910 10449539

[B41] Economou A Following the leader: bacterial protein export through the Sec pathway Trends in Microbiology 1999 7 315 320 10.1016/S0966-842X(99)01555-3 10431204

[B42] Manz R Assenmacher M Pflüger E Miltenyi S Radbruch A Analysis and sorting of live cells according to secreted molecules, relocated to a cell-surface affinity matrix Proceedings of the National Academy of Sciences of the United States of America 1995 92 1921 10.1073/pnas.92.6.1921 7892200 PMC42394

[B43] Frykman S Srienc F Quantitating secretion rates of individual cells: design of secretion assays Biotechnology and bioengineering 1998 59 214 226 10.1002/(SICI)1097-0290(19980720)59:2<214::AID-BIT9>3.0.CO;2-K 10099332

[B44] Müller S Nebe von Caron G Functional single cell analyses: flow cytometry and cell sorting of microbial populations and communities FEMS Microbiology Reviews 2010 34 554 587 20337722 10.1111/j.1574-6976.2010.00214.x

[B45] Feldhaus M Siegel R Flow cytometric screening of yeast surface display libraries METHODS IN MOLECULAR BIOLOGY-CLIFTON THEN TOTOWA 2004 263 311 332 10.1385/1-59259-773-4:311 14976374

[B46] Mattanovich D Borth N Applications of cell sorting in biotechnology Microbial Cell Factories 2006 5 12 10.1186/1475-2859-5-12 16551353 PMC1435767

[B47] Lewis G Taylor IW Nienow AW Hewitt CJ The application of multi-parameter flow cytometry to the study of recombinant Escherichia coli batch fermentation processes Journal of Industrial Microbiology and Biotechnology 2004 31 311 322 10.1007/s10295-004-0151-8 15249970

[B48] Want A Thomas ORT Kara B Liddell J Hewitt CJ Studies related to antibody fragment (Fab) production in Escherichia coli W3110 fed-batch fermentation processes using multiparameter flow cytometry Cytometry Part A 2009 75 148 154 10.1002/cyto.a.20683 19051239

[B49] Hewitt CJ Nebe-Von Caron G Axelsson B McFarlane CM Nienow AW Studies related to the scale-up of high-cell-density E. coli fed-batch fermentations using multiparameter flow cytometry: effect of a changing microenvironment with respect to glucose and dissolved oxygen concentration Biotechnology and bioengineering 2000 70 381 390 10.1002/1097-0290(20001120)70:4<381::AID-BIT3>3.0.CO;2-0 11005920

[B50] Hewitt CJ Nebe-Von Caron G Nienow AW McFarlane CM The use of multi-parameter flow cytometry to compare the physiological response of Escherichia coli W3110 to glucose limitation during batch, fed-batch and continuous culture cultivations Journal of biotechnology 1999 75 251 264 10.1016/S0168-1656(99)00168-6 10553662

[B51] Lara AR Galindo E Ramírez OT Palomares LA Living with heterogeneities in bioreactors Molecular biotechnology 2006 34 355 381 10.1385/MB:34:3:355 17284782

[B52] Sitton G Srienc F Mammalian cell culture scale-up and fed-batch control using automated flow cytometry Journal of biotechnology 2008 135 174 180 10.1016/j.jbiotec.2008.03.019 18490070

[B53] Laflamme C Ho J Veillette M de Latrémoille MC Verreault D Meriaux A Duchaine C Flow cytometry analysis of germinating Bacillus spores, using membrane potential dye Archives of microbiology 2005 183 107 112 10.1007/s00203-004-0750-9 15611861

